# Demethoxycurcumin Alleviates Lipopolysaccharide‐Induced Acute Lung Injury via Nrf2‐Mediated Anti‐Inflammation and Ferroptosis Inhibition

**DOI:** 10.1155/mi/9154680

**Published:** 2026-09-09

**Authors:** Jing Tang, Qiuni Zhao, Jue Liu, Chuangxin Zheng, Zhenbiao He, Ji Xiao

**Affiliations:** ^1^ Department of Anesthesiology, NHC Key Laboratory of Birth Defect for Research and Prevention (Hunan Provincial Maternal and Child Health Care Hospital), Changsha, Hunan, 410008, China, hunanfy.com; ^2^ Department of Anesthesiology, The Second Affiliated Hospital of University of South China, Hengyang, Hunan, 421001, China, usc.edu.cn; ^3^ Department of Anesthesiology, The Affiliated Cancer Hospital of Xiangya School of Medicine, Central South University, Changsha 410013, Hunan, China, csu.edu.cn

**Keywords:** demethoxycurcumin, ferroptosis, inflammation, Keap1/Nrf2 pathway, oxidative stress, sepsis-induced acute lung injury

## Abstract

**Background:**

Sepsis‐induced acute lung injury (SI‐ALI) represents a life‐threatening condition driven by dysregulated immune responses and redox imbalance. Demethoxycurcumin (DMC), a bioactive analog of curcumin, exhibits marked anti‐inflammatory and free radical‐scavenging capacities. Despite its potential, the precise molecular pathways through which DMC mitigates SI‐ALI pathogenesis have yet to be fully elucidated.

**Methods:**

SI‐ALI was induced in mice through lipopolysaccharide (LPS) administration. Lung injury was assessed by histopathological analysis and measurement of wet‐to‐dry (W/D) weight ratios. Oxidative stress markers were quantified using ELISA, and inflammatory cytokine mRNA levels were analyzed by RT‐PCR. Ferroptosis‐related proteins and Nrf2 pathway activation were evaluated through Western blotting and immunofluorescence. Mitochondrial ultrastructure was examined via transmission electron microscopy (TEM). The involvement of the Nrf2 pathway was further confirmed by using the Nrf2 inhibitor ML385.

**Results:**

DMC treatment significantly alleviated lung injury in a dose‐dependent manner, improving histopathology and reducing W/D weight ratios. It attenuated inflammation by suppressing IL‐6, TNF‐α, and IL‐1β expression and restored oxidative balance by enhancing glutathione (GSH) levels while decreasing malondialdehyde (MDA). Moreover, DMC upregulated glutathione peroxidase 4 (GPX4) and SLC7A11, downregulated cyclooxygenase‐2 (COX2) and ACSL4, reduced nonheme iron and 4‐hydroxynonenal (4‐HNE) production, and improved mitochondrial morphology, collectively demonstrating its inhibitory effects on ferroptosis. Nrf2 inhibition partially reversed these protective effects, underscoring the central role of the Nrf2 pathway in DMC’s mechanism of action.

**Conclusion:**

DMC ameliorates SI‐ALI by mitigating inflammation and ferroptosis through Nrf2 pathway activation, suggesting a potential therapeutic strategy for SI‐ALI treatment.

## 1. Introduction

Sepsis‐induced acute lung injury (SI‐ALI) is a common and severe complication in septic patients, characterized by significant damage to alveolar epithelial and capillary endothelial cells [1, 2]. This damage compromises the integrity of the alveolar‐capillary barrier, resulting in pulmonary edema, decreased oxygen levels, and impaired gas exchange. In advanced stages, SI‐ALI can progress to acute respiratory distress syndrome (ARDS), a critical condition with limited therapeutic options, associated with a reported 90‐day mortality rate of ~35.5% [3]. Despite advancements in critical care management, the lack of specific therapeutic measures for ARDS highlights the pressing need to investigate the underlying mechanisms of SI‐ALI.

Among the newly recognized mechanisms, ferroptosis, a form of iron‐dependent cell death, has been identified as a contributing factor in the development of SI‐ALI [4]. Ferroptosis is characterized by the buildup of reactive oxygen species (ROS) and lipid peroxides due to a compromised antioxidant system, with a key role played by reduced glutathione peroxidase 4 (GPX4) activity [5]. In experimental models of SI‐ALI, ferroptosis markers, such as elevated levels of malondialdehyde (MDA) and iron ions, have been observed, alongside decreased expression of GPX4 [6]. Furthermore, pharmacological inhibition of ferroptosis using agents such as liproxstatin‐1 has been shown to attenuate pulmonary histopathological changes and reduce inflammatory responses in murine models, underscoring the pivotal role of ferroptosis in SI‐ALI pathophysiology [7, 8].

The mechanisms driving ferroptosis in SI‐ALI are intricate and involve various metabolic pathways, including iron homeostasis, amino acid metabolism, lipid peroxidation, and glutathione (GSH) synthesis [4, 9]. Recent studies have highlighted the involvement of the Keap1‐Nrf2 signaling pathway in ferroptosis regulation, with the Nrf2/Slc7a11 axis exhibiting a protective role in mitigating oxidative damage and inflammation in acute lung injury [10]. These insights suggest that targeting ferroptosis through therapeutic strategies that modulate oxidative stress and inflammatory responses could offer promising new treatment options for SI‐ALI.

Demethoxycurcumin (DMC), a natural bioactive derivative of curcumin, possesses strong anti‐inflammatory, antioxidant, and antiproliferative activities [11]. These bioactivities make DMC a promising candidate for SI‐ALI intervention, as oxidative stress and inflammation dominate the pathological progression of this disease [9]. Accumulating studies have demonstrated that DMC elicits antioxidant effects via activating the Keap1‐Nrf2 pathway, a core endogenous defense system against oxidative stress and ferroptosis [12, 13]. While curcumin and its analogs can relieve inflammation and oxidative stress in ALI models, current research on DMC mainly concentrates on its anti‐inflammatory capacity [14, 15]. To our knowledge, systematic studies regarding the regulatory effect of DMC on ferroptosis in SI‐ALI are still limited. Moreover, direct evidence connecting DMC intervention, Nrf2 pathway, and ferroptosis is currently absent. Since ferroptosis is a pivotal pathological driver of SI‐ALI, deciphering the interplay between DMC, the Nrf2 pathway, and ferroptosis will fill the above research gaps and offer new preclinical support for the clinical translation of DMC.

Based on the vital role of ferroptosis in SI‐ALI, we hypothesize that DMC protects against SI‐ALI through modulating ferroptosis‐related signaling. Mechanistically, DMC may activate the Nrf2 pathway to inhibit lipid peroxidation, thereby attenuating ferroptosis‐induced oxidative damage and inflammatory responses.

## 2. Materials and Methods

### 2.1. Reagents and Materials

Lipopolysaccharide (LPS; L4524, sourced from *E. coli* 055: B5) and DMC were purchased from Sigma Aldrich (St. Louis, MO, USA). Superoxide dismutase (SOD), GSH, and MDA assay kits were obtained from Beyotime Biotechnology (Shanghai, China). Iron assay kit (A039‐2‐1) was purchased from Nanjing Jiancheng Bioengineering Institute (Nanjing, China). Antibodies for Western blotting, including those against GPX4, SLC7A11, ACSL4, cyclooxygenase‐2 (COX2), HO‐1, NQO1, Keap1, and Nrf2, were supplied by BOSTER (Wuhan, China), while the anti‐4‐hydroxynonenal (4‐HNE) antibody was purchased from Abcam (Cambridge, UK). RT‐PCR primers were synthesized by Sangon Biotech (Shanghai, China). All other reagents were sourced from commercial suppliers, unless otherwise.

### 2.2. Animal Model

Male C57BL/6 mice (8 weeks old, weighing 20–25 g) were used in accordance with the National Institutes of Health (NIH) guidelines for the ethical care and use of laboratory animals. The animals were kept in a pathogen‐free environment with a 12‐h light/dark cycle and had unrestricted access to water and food. All mice were randomly assigned to different experimental groups using a random number table to avoid selection bias, with six mice in each group (*n* = 6). The experimental groups were divided as follows: control group, LPS model group, LPS + 25 mg/kg DMC group, LPS + 50 mg/kg DMC group, and LPS + 100 mg/kg DMC group. For subsequent Nrf2 inhibitor intervention experiments, an additional LPS + DMC + ML385 group was established, with six mice per group uniformly. The sample size of *n* = 6 was determined according to our previous animal experiments and published literatures on lung injury models, which meets the requirements for statistical analysis.

ALI was induced by intraperitoneal injection of LPS (15 mg/kg body weight, dissolved in sterile saline), while control mice were administered an equal volume of saline. To investigate the protective effects of DMC, mice were intragastrically administered DMC at doses of 0, 25, 50, or 100 mg/kg body weight, selected based on previous studies indicating that 60 mg/kg represents the upper limit of the effective range without overt toxicity [11]. After the treatment, the mice were euthanized under deep anesthesia, and lung tissues were collected for biochemical, histological, and molecular analyses. All procedures were conducted with the aim of minimizing animal discomfort throughout the experiment.

### 2.3. Hematoxylin and Eosin (H&E) Staining

Lung tissues were initially fixed in 10% neutral‐buffered formalin, followed by paraffin embedding and sectioning into 4‐µm‐thick slices. The sections were deparaffinized, rehydrated, and stained with hematoxylin for 5 min and then differentiated with 1% acid alcohol and blued using Scott’s tap water substitute. Eosin staining was applied for 1–3 min, followed by dehydration, clearing in xylene, and mounting with coverslips. Histopathological evaluation of the stained lung sections was carried out independently by two blinded experienced pathologists, who were unaware of the experimental groups. Key features, such as the integrity of alveolar structures, infiltration of inflammatory cells, thickening of alveolar septa, and hemorrhage, were assessed. The results from the two pathologists were compared for consistency, and any differences were resolved through consensus. Lung injury was quantitatively scored using a standardized histopathological scoring system [16].

### 2.4. Lung Wet‐to‐Dry (W/D) Weight Ratio

The lung W/D weight ratio was measured to evaluate the extent of pulmonary edema. The left lung was carefully excised and immediately weighed to obtain the wet weight. Afterward, the lung tissue was placed in a drying oven set at 60°C for 72 h, ensuring complete dehydration. Once dried, the lung was reweighed to determine the dry weight. The W/D weight ratio, calculated by dividing the wet weight by the dry weight, serves as a reliable quantitative indicator of tissue edema, reflecting the degree of fluid accumulation within the lung tissue.

### 2.5. ELISA for MDA and GSH

The levels of MDA and GSH in lung tissue homogenates were quantified using commercially available ELISA kits, following the manufacturer’s instructions. Lung tissues were first homogenized, and the resulting homogenates were processed for analysis. Absorbance readings at specific wavelengths were obtained using a microplate reader (Thermo Fisher Scientific, USA). The optical density (OD) values for each sample were compared to standard curves generated for each biomarker. The concentrations of GSH and MDA in the samples were subsequently determined from the standard curves, providing a quantitative measure of oxidative stress markers and cellular damage in lung tissues.

### 2.6. Quantitative RT‐PCR

Total RNA was extracted from lung tissues using TRIzol reagent (Invitrogen, USA). The concentration and purity of RNA were measured using a NanoDrop spectrophotometer, ensuring that the extracted RNA met the required quality standards for downstream applications. Complementary DNA (cDNA) was synthesized from the extracted RNA using a reverse transcription kit (Takara, Japan), following the manufacturer’s recommended protocols for optimal cDNA yield. qRT‐PCR was then performed using SYBR Green Master Mix (Applied Biosystems, USA) on a QuantStudio 5 Real‐Time PCR System to quantify the mRNA levels of target gene, including IL‐6, TNF‐α, IL‐1β, HO‐1, and NQO1. The relative expression levels of these genes were normalized to the housekeeping gene glyceraldehyde 3‐phosphate dehydrogenase (GAPDH) to account for potential variations in RNA quantity and quality. The 2^−ΔΔCt^ method was used to calculate the fold change in gene expression, allowing for the comparison of inflammatory responses across different experimental conditions.

### 2.7. Western Blot Analysis

Lung tissue proteins were carefully extracted using RIPA buffer supplemented with protease and phosphatase inhibitors to prevent enzymatic degradation and dephosphorylation during the extraction process. The protein concentration in the lysates was quantified using a bicinchoninic acid (BCA) assay kit, following the manufacturer’s instructions for accurate measurement. Equal quantities of protein (30 µg) were then subjected to electrophoresis on SDS‐PAGE to separate the proteins according to their molecular weight. After separation, the proteins were transferred onto PVDF membranes using a semidry transfer system. To block nonspecific binding sites, the membranes were incubated with a blocking solution containing 5% nonfat milk for 1 h at room temperature. Subsequently, the membranes were incubated overnight at 4°C with specific primary antibodies against GPX4, SLC7A11, COX2, ACSL4, HO‐1, NQO1, Keap1, and Nrf2 to target the proteins of interest. After washing, the membranes were incubated with HRP‐conjugated secondary antibodies for 1 h at room temperature. Protein bands were visualized through chemiluminescence using an enhanced chemiluminescence detection system (Bio‐Rad, USA), providing clear and sensitive detection of the target proteins.

### 2.8. Immunofluorescence Staining

Paraffin‐embedded lung tissue sections (5 µm thick) were first deparaffinized in xylene, followed by a rehydration process using graded ethanol solutions. Antigen retrieval was performed to enhance the accessibility of target epitopes, typically by heating the sections in a citrate buffer. After the antigen retrieval step, the sections were incubated overnight at 4°C with a primary antibody specific for 4‐HNE. Following primary antibody incubation, the sections were exposed to a secondary antibody conjugated with Alexa Fluor 594 to enable fluorescence detection. To visualize cell nuclei, the sections were counterstained with DAPI, which binds to DNA. Finally, fluorescence images of the stained tissue sections were acquired using a fluorescence microscope (Leica, Germany), allowing for the localization and evaluation of 4‐HNE expression within the lung tissues.

### 2.9. Transmission Electron Microscopy (TEM)

Small pieces of lung tissue (~1 mm^3^) were first fixed in a solution of 2.5% glutaraldehyde at 4°C to preserve cellular integrity and ensure optimal preservation of subcellular components. Following fixation, tissues underwent postfixation in 1% osmium tetroxide, which serves to stabilize lipid membranes and enhance contrast for electron microscopy. The samples were then subjected to a dehydration process in a graded series of ethanol concentrations, ensuring proper removal of water and maintaining the ultrastructure. Subsequently, the tissues were embedded in epoxy resin, providing a rigid matrix that supports the delicate structures during sectioning. Ultrathin sections (typically 50–70 nm thick) were prepared using a diamond knife. These sections were then stained with uranyl acetate and lead citrate to improve contrast, allowing for the fine details of the mitochondrial architecture to be clearly visualized. The prepared samples were examined under a transmission electron microscope (JEOL, Japan), which enabled high‐resolution imaging of the mitochondrial morphology, facilitating detailed analysis of their structural integrity and potential alterations.

### 2.10. Statistical Analysis

The data are expressed as the mean ± standard deviation (SD), providing a summary measure of the central tendency and variability. All statistical analyses were carried out using GraphPad Prism 9.0 software. For comparisons involving more than two groups, a one‐way analysis of variance (ANOVA) was employed to assess overall differences between groups. Following the ANOVA, the Tukey’s post hoc test was applied to perform pairwise comparisons between each group, ensuring a comprehensive evaluation of all possible differences. Statistical significance was determined by a *p*‐value of less than 0.05, indicating a threshold below which the null hypothesis was rejected, and differences were considered statistically meaningful.

## 3. Results

### 3.1. DMC Attenuates LPS‐Induced Inflammation and ALI

**Figure 1:**
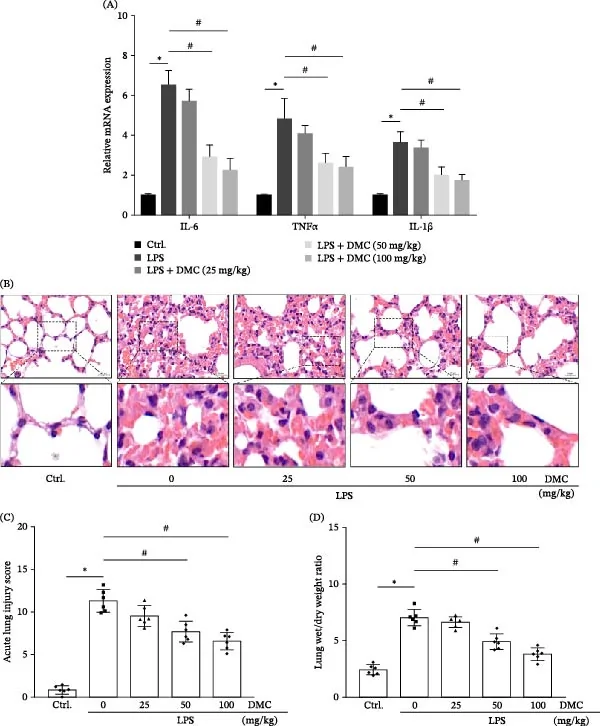
Dose‐dependent protective effects of DMC on LPS‐induced acute lung injury in mice. (A) Relative mRNA expression levels of proinflammatory cytokines IL‐6, TNF‐α, and IL‐1β in lung tissues. (B) Representative images of hematoxylin and eosin (H&E)–stained lung sections showing histopathological changes following treatment with different doses of DMC (0, 25, 50, and 100 mg/kg). (C) Quantitative analysis of acute lung injury scores, demonstrating significant reductions with increasing doses of DMC. (D) Lung wet‐to‐dry (W/D) weight ratio. Data are presented as mean ± SD (*n* = 6 per group).  ^∗^ *p* < 0.05 compared to the Ctrl. group and ^#^ *p* < 0.05 compared to the LPS‐only group.

Exposure to LPS significantly upregulated the mRNA expression of key proinflammatory cytokines, including IL‐6, TNF‐α, and IL‐1β, in lung tissues, indicating a robust pulmonary inflammatory response. The administration of DMC demonstrated a dose‐dependent reduction in LPS‐induced pulmonary inflammation (Figure 1A). Histopathological evaluation using H&E staining showed significant restoration of lung tissue structure in the DMC‐treated groups, particularly at doses of 50 and 100 mg/kg, when compared to the LPS‐only control group (Figure 1B). These improvements in lung architecture were marked by reduced inflammatory cell infiltration and lessened alveolar damage, suggesting that DMC effectively mitigates the pathological consequences of ALI. Quantitative assessments further supported the protective effects of DMC. ALI severity scores, which reflect the extent of lung damage, were notably lower in the DMC‐treated groups. Similarly, the lung W/D weight ratios, an important indicator of pulmonary edema and fluid accumulation, were significantly decreased after DMC treatment, with the most substantial benefits observed at the 50 mg/kg dose (Figure 1C,D). These findings underscore the potential of DMC as an effective therapeutic agent in the management of LPS‐induced ALI.

### 3.2. Modulation of Ferroptosis by DMC

**Figure 2:**
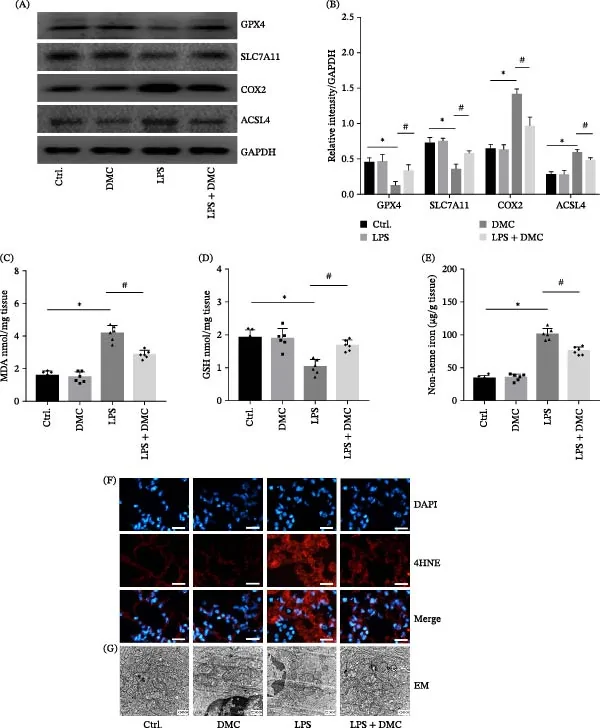
DMC regulates ferroptosis markers and improves mitochondrial morphology in lung epithelial cells. (A) Western blot analysis of GPX4, SLC7A11, COX2, and ACSL4 protein levels in lung tissues. (B) Semiquantitative analysis of GPX4, SLC7A11, COX2, and ACSL4 protein expression levels. (C–E) Quantitative analyses of MDA, GSH, and nonheme iron. (F) Immunofluorescence staining for 4‐HNE in lung tissues. (G) TEM images of lung epithelial cells, showing improvements in mitochondrial morphology. Data are presented as mean ± SD (*n* = 6 per group).  ^∗^ *p* < 0.05 compared to the Ctrl. group and ^#^ *p* < 0.05 compared to the LPS‐only group.

To assess the effect of DMC on ferroptosis, we first investigated its impact on key ferroptotic markers. Western blot analysis revealed that DMC treatment significantly reduced the expression of COX2 and ACSL4 while simultaneously upregulating the expression of GPX4 and SLC7A11 in lung tissues (Figure 2A,B). Moreover, DMC treatment resulted in increased levels of GSH, as well as decreased MDA and nonheme iron levels (Figure 2C–E). Immunofluorescence staining demonstrated a marked reduction in the levels of 4‐HNE, a well‐established marker of lipid peroxidation and a key indicator of ferroptosis (Figure 2F). The significant decrease in 4‐HNE production underscores the ability of DMC to mitigate lipid peroxidation, which is a crucial event in the onset and progression of ferroptosis. Finally, TEM provided compelling ultrastructural evidence of DMC’s protective effects on mitochondria. DMC treatment resulted in significant improvements in mitochondrial morphology, characterized by a reduction in mitochondrial shrinkage and the restoration of membrane integrity‐two hallmark features of ferroptosis (Figure 2G). Collectively, these findings demonstrate that DMC effectively attenuates ferroptosis in the context of LPS‐induced ALI, highlighting its potential as a therapeutic agent to prevent ferroptosis‐associated damage in lung tissues.

### 3.3. Regulation of the Nrf2 Pathway by DMC

**Figure 3:**
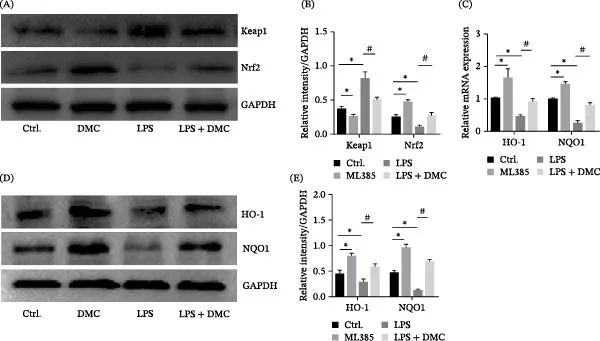
DMC modulates the Keap1/Nrf2 pathway in LPS‐induced lung injury. (A, D) Western blot analysis of Keap1, Nrf2, HO‐1, and NQO1 protein levels in lung tissues. (B, E) Semiquantitative analysis of Keap1, Nrf2, HO‐1, and NQO1 protein expression. (C) Relative mRNA expression levels of HO‐1 and NQO1 in lung tissues. Data are presented as mean ± SD (*n* = 6 per group).  ^∗^ *p* < 0.05 compared to the control group and ^#^ *p* < 0.05 compared to the LPS‐only group.

The Nrf2 signaling pathway plays a pivotal role in regulating oxidative stress and activating cellular defense mechanisms, particularly in the context of ferroptosis and lung injury. To explore the effect of DMC on the Nrf2 pathway, we examined its influence on key components of this signaling axis. Western blot analysis revealed a significant disruption of the Keap1/Nrf2 pathway following LPS exposure in lung tissues. Specifically, LPS treatment resulted in elevated Keap1 expression, which accelerates the degradation of Nrf2, while concurrently decreasing Nrf2 levels. This dysregulation impairs the lung’s antioxidant defenses. In contrast, DMC treatment effectively reversed these changes, significantly reducing Keap1 expression and increasing Nrf2 levels (Figure 3A,B). Consistent with this rescue, DMC also upregulated the mRNA and protein expression of the canonical Nrf2 downstream target genes HO‐1 and NQO1 in LPS‐challenged lungs (Figure 3C–E). These results suggest that DMC restores the proper function of the Keap1/Nrf2 axis, thereby enhancing the cellular defense mechanisms and providing protection against LPS‐induced damage.

### 3.4. Role of Nrf2 in the Regulation of Ferroptosis by DMC

**Figure 4:**
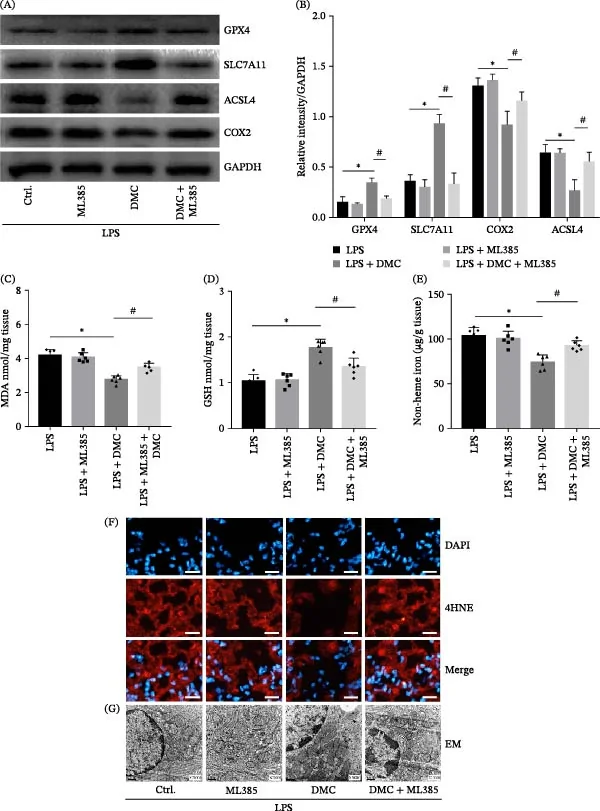
Nrf2 inhibition partially reverses the protective effects of DMC on ferroptosis. (A) Western blot analysis of GPX4, SLC7A11, COX2, and ACSL4 protein levels in lung tissues. (B) Semiquantitative analysis of GPX4, SLC7A11, COX2, and ACSL4 protein expression levels. (C–E) Quantitative analyses of MDA, GSH, and nonheme iron. (F) Immunofluorescence staining for 4‐HNE in lung tissues. (G) TEM images of lung epithelial cells, showing improvements in mitochondrial morphology. Data are presented as mean ± SD (*n* = 6 per group).  ^∗^ *p* < 0.05 compared to the LPS‐only group and ^#^ *p* < 0.05 compared to the LPS + DMC group.

Nrf2 drives the expression of various antioxidant genes that help mitigate cellular damage, including lipid peroxidation, which is a hallmark of ferroptosis. To explore the impact of the Nrf2 pathway on ferroptosis in the context of DMC’s effects on LPS‐induced ALI, we utilized the Nrf2 inhibitor ML385. Treatment with ML385 partially attenuated the protective effects of DMC on LPS‐ALI. Specifically, ML385 treatment reduced the ability of DMC to suppress COX2 and SLC7A11 expression and enhance GPX4 and SLC7A11 expression (Figure 4A,B). In addition, ML385 treatment resulted in decreased levels of GSH, as well as increased MDA and nonheme iron levels (Figure 4C–E), further suggesting a disruption of antioxidant defense mechanisms. Immunofluorescence staining revealed that ML385 partially reversed the inhibitory effects of DMC on 4‐HNE production, a marker of lipid peroxidation associated with ferroptosis (Figure 4F). Moreover, TEM demonstrated that ML385 partially reversed the mitochondrial morphological improvements induced by DMC, such as the reduction in mitochondrial shrinkage and the restoration of membrane integrity (Figure 4G). These findings highlight the critical role of Nrf2 in mediating the regulatory effects of DMC on ferroptosis.

### 3.5. Role of Nrf2 in the Regulation of ALI by DMC

**Figure 5:**
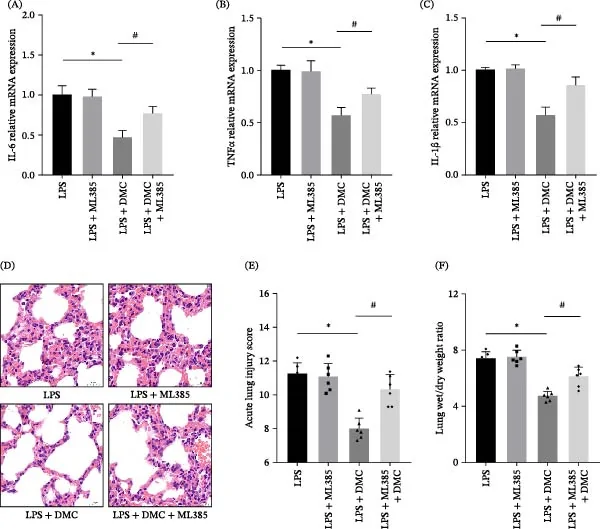
Nrf2 inhibition attenuates the anti‐inflammatory and lung‐protective effects of DMC. (A–C) Relative mRNA expression levels of proinflammatory cytokines IL‐6, TNF‐α, and IL‐1β in lung tissues. (D) Representative images of H&E‐stained lung sections showing histopathological changes. (E) Quantitative analysis of acute lung injury scores. (F) Lung wet‐to‐dry (W/D). Data are expressed as mean ± SD (*n* = 6 per group).  ^∗^ *p* < 0.05 compared to the LPS‐only group and ^#^ *p* < 0.05 compared to the LPS + DMC group.

Nrf2 activation plays a critical role in safeguarding lung tissues from injury by modulating inflammatory pathways. To investigate the involvement of the Nrf2 pathway in the protective effects of DMC against LPS‐induced ALI, we utilized the Nrf2 inhibitor ML385. Treatment with ML385 partially reversed the anti‐inflammatory and lung‐protective effects of DMC. Specifically, ML385 diminished the ability of DMC to suppress the expression of LPS‐induced proinflammatory cytokines (Figure 5A–C), alleviate histopathological lung damage (Figure 5D), reduce acute lung injury scores (Figure 5E), and improve the lung W/D weight ratios (Figure 5F). These results provide further evidence that Nrf2 activation is a key mechanism through which DMC exerts its protective effects against LPS‐induced ALI.

## 4. Discussion

SI‐ALI remains a major clinical challenge, primarily due to its high mortality rate and the lack of effective treatment options [2]. Despite significant advancements in critical care, managing SI‐ALI continues to be difficult, as therapeutic interventions are limited and often insufficient to address the complex pathophysiology of the condition. In this study, we provide compelling evidence that DMC offers significant protective effects against LPS‐induced ALI in a murine model. Our findings offer important insights into the underlying molecular mechanisms that contribute to the therapeutic potential of DMC, emphasizing its ability to modulate the Nrf2 signaling pathway, a critical regulator of cellular responses to inflammation and ferroptosis. This research paves the way for further exploration into DMC’s potential as a novel treatment strategy for SI‐ALI and other related inflammatory diseases.

These results support the notion that DMC effectively mitigates LPS‐induced inflammation, consistent with the anti‐inflammatory properties reported for other curcumin derivatives [17, 18]. By inhibiting the overproduction of key inflammatory mediators, DMC helps attenuate the harmful effects of the inflammatory response, preserving the integrity of the alveolar‐capillary barrier and ultimately preventing further lung damage. This highlights the potential of DMC as a therapeutic agent capable of modulating the inflammatory response in SI‐ALI.

Oxidative stress is another critical driver of SI‐ALI, contributing to lipid peroxidation, cellular damage, and the amplification of inflammatory responses. In this study, DMC demonstrated a strong capacity to restore the antioxidant balance in LPS‐induced ALI. Specifically, DMC treatment increased the levels of key antioxidant enzymes, including GSH, while simultaneously reducing the accumulation of MDA, a marker of lipid peroxidation. By decreasing MDA levels, DMC helps to mitigate the harmful effects of lipid peroxidation, which is a major contributor to lung injury in the context of oxidative stress [19]. Furthermore, the ability of DMC to enhance the lung’s antioxidant defense system aligns with its previously reported function as a potent scavenger of ROS, further strengthening its potential as a therapeutic agent in oxidative stress–related disorders [20, 21].

Ferroptosis is an iron‐dependent cell death modality driven by excessive lipid peroxidation, which acts as a critical pathological executor in SI‐ALI [22]. As classic terminal products of lipid peroxidation, MDA and 4‐HNE were detected in this study to reflect the degree of ferroptosis in lung tissues. Our data confirmed that DMC could reverse the abnormal expression of core ferroptosis regulatory proteins GPX4, SLC7A11, ACSL4, and COX2; restore intracellular GSH homeostasis; and reduce iron overload, thereby alleviating ferroptotic damage in alveolar epithelial cells. Mitochondrial morphological improvement further verified the antiferroptosis effect of DMC at the subcellular level.

The Nrf2 pathway plays a pivotal role in regulating cellular responses to oxidative stress and ferroptosis, acting as a crucial sensor and mediator of cellular defense mechanisms [23, 24]. Upon activation, Nrf2 translocates to the nucleus and induces the transcription of a wide array of cytoprotective genes involved in antioxidant defense, including those regulating GSH synthesis and ROS detoxification [25]. In this study, we demonstrate that DMC effectively activates the Nrf2 pathway by inhibiting Keap1, a negative regulator of Nrf2, and by promoting the expression of Nrf2 and its downstream target genes (HO‐1 and NQO1). In the cytoplasm, Keap1 continuously binds to Nrf2 and mediates its ubiquitination and degradation under physiological conditions. LPS stimulation disrupted redox balance and further enhanced Keap1 expression, leading to sustained Nrf2 degradation and impaired cellular antioxidant defense. DMC treatment suppressed Keap1 expression, stabilized Nrf2 protein, and triggered the activation of the Nrf2 signaling cascade. The upregulation of HO‐1 and NQO1 further enhanced the antioxidant capacity of lung tissues, forming a complete endogenous protective network.

To further validate the functional importance of this pathway, we employed ML385, a selective Nrf2 inhibitor. Blockade of Nrf2 significantly attenuated the protective effects of DMC, as evidenced by increased inflammatory cytokine levels, elevated oxidative stress markers, and enhanced indicators of ferroptosis. Moreover, ML385 treatment abolished DMC’s ability to improve lung histopathology and mitochondrial integrity, underscoring the central role of Nrf2 in mediating DMC’s therapeutic actions. These findings provide strong evidence that Nrf2 activation is both necessary and sufficient for DMC‐mediated protection in LPS‐induced ALI.

Despite the promising results, several limitations of this study should be acknowledged. First, while we have demonstrated that DMC modulates the Nrf2 pathway, the precise molecular interactions between DMC and key components of this axis—such as Keap1, Nrf2, and antioxidant response elements (ARE)—require further investigation using in vitro models and molecular docking studies. Second, the long‐term safety and pharmacokinetic profile of DMC remain to be fully evaluated. Although our data support its efficacy in a preclinical setting, comprehensive toxicological assessments and clinical trials are essential to determine its safety, optimal dosing, and therapeutic window in humans. Lastly, the potential for synergistic effects between DMC and other agents targeting oxidative stress or ferroptosis warrants exploration. Combination therapies may enhance therapeutic efficacy and expand the clinical applicability of DMC in managing complex inflammatory diseases such as SI‐ALI. In addition, this current work mainly focuses on in vivo animal experiments to clarify the macroscopic protective effects of DMC. Further detection of lipid ROS and exploration of Nrf2 nuclear translocation will be performed in our subsequent in vitro cell experiments, which will help to elaborate the molecular mechanism of DMC more comprehensively at the cellular level.

## 5. Conclusion

In summary, our study highlights the therapeutic potential of DMC in mitigating LPS‐induced ALI through anti‐inflammatory and ferroptosis‐modulating properties. By modulating the Nrf2 pathway, DMC effectively restores redox balance, reduces lipid peroxidation, and preserves mitochondrial function. These mechanistic insights underscore the promising role of DMC as a novel therapeutic strategy for SI‐ALI, offering a potential new approach to addressing this critical condition.

## Funding

This study was supported by grants from the Hunan Provincial Natural Science Foundation of China (Grants 2024JJ5243 and 2025JJ80655), the Scientific Research Project of Hunan Province Chinese Medicine (Grant D2022103), and the Hunan Cancer Hospital Climb Plan (Grant 2023NSFC‐A005).

## Disclosure

All authors gave their content for publication.

## Ethics Statement

All experimental procedures were performed in strict accordance with the guidelines for the care and use of laboratory animals, as outlined by the National Institutes of Health (NIH). The experimental protocols were reviewed and approved by the Ethics Committee for Experimental Animals of the Affiliated Cancer Hospital, Xiangya School of Medicine, Central South University, as well as the Ethics Committee of Hunan Provincial Maternal and Child Health Care Hospital.

## Conflicts of Interest

The authors declare no conflicts of interest.

## Data Availability

The datasets generated and/or analyzed during the present study are available from the corresponding author upon reasonable request.
